# Entrepreneurship and entrepreneurial opportunities in the food value chain

**DOI:** 10.1038/s41538-019-0039-7

**Published:** 2019-04-16

**Authors:** Andreas Kuckertz, Sebastian Hinderer, Patrick Röhm

**Affiliations:** Entrepreneurship Research Group, University of Hohenheim, Institute of Marketing & Management, Stuttgart, Germany

**Keywords:** Economics, Economics

## Abstract

Successful entrepreneurs exploit entrepreneurial opportunities to establish their enterprises. The food industry is a setting in which many such opportunities exist. To shed light on those specific entrepreneurial opportunities, we combine a five-step model of the food value chain ranging from agriculture to consumption with classic economic sources of entrepreneurial opportunities, that is, changes in supply, changes in demand, exogenous shocks, or informational asymmetries prevalent in the market. We proxy for entrepreneurial opportunities by shedding light on where start-up investors assign their capital in the food value chain. Data drawn from the Dow Jones VentureSource database is recoded to illustrate this investment behavior. Consequently, we are able to (a) illustrate where in the food value chain in particular investors perceive the most upside potential and (b) construct a map of the entrepreneurial opportunity space in the food industry. The results are informative, particularly for aspiring food entrepreneurs searching for entrepreneurial opportunities and aiming to raise funding for their entrepreneurial endeavors.

## Introduction

Start-ups establishing innovative business models now receive a great deal of attention. However, both academic research and the media in general tend to focus only on a minority of start-ups, and particularly those pursuing a business model based on the potential of digitization. This creates a distorted picture of the reality of entrepreneurial activity, as whole sectors can be overlooked; for instance, despite its apparent potential, innovative entrepreneurial behavior in the food industry is rarely reported. Astonishingly, the Global Entrepreneurship Monitor,^1^ an international study comparing the state of entrepreneurship in numerous economies worldwide every year, provides no information at all on entrepreneurial activity in this important industry. Other prominent studies on entrepreneurial activity^2^ also ignore the food sector.

This lack of scholarly and media attention continues despite the three largest markets for food alone (i.e., USA, China and India) creating combined revenues of USD 1,274,239.68 million in 2017.^3^ The food sector is clearly significant for economies worldwide, and at the same time, the industry faces huge challenges such as food supply,^4^ food security^5,6^ or food waste^7^ that might offer interesting opportunities for aspiring entrepreneurs developing innovative solutions to those pressing issues. Against this background, one aim of this perspective paper is to provide an evidence-based view on what start-ups contribute to the food industry and where opportunities for future entrepreneurial activity might lie. To do so, we provide an analysis of data of investment activity related to a five-step conceptualization of the food value chain. Doing so illuminates patterns that might indicate where start-up investors perceive the most promising entrepreneurial opportunities to be. Additionally, we combine theoretically grounded sources of entrepreneurial opportunities that are well established in the economic literature with our conceptualization of the food value chain to create a map of the entrepreneurial opportunity space for food entrepreneurs.

## Entrepreneurship and entrepreneurial opportunities

Entrepreneurship, which we understand as the creation of growth-oriented organizations to exploit mostly economic opportunities^8^ is of utmost importance for economies of every type.^2^ Innovation-driven economies, such as those of the USA, Japan, and most European countries benefit from new jobs created by entrepreneurs and additional economic growth. Less-developed economies, such as factor-driven and efficiency-driven economies additionally face the potential of formalizing informal economic activity and reducing poverty. Policy makers have recognized that the food industry can contribute significantly to these goals,^9^ for instance, by establishing the Food Knowledge and Innovation Community within the European Institute of Innovation and Technology^10^ that aims to empower entrepreneurs and others to provide novel solutions to pressing issues in the food industry.

The concept of entrepreneurial opportunity is central to entrepreneurship^11^ and economic theory suggests a number of sources of such opportunities that could help structure the food industry (Table 1). First, whenever customer demand changes, opportunities for entrepreneurs to cater to these new demands emerge. For instance, consumers’ increasing awareness of healthy or sustainably produced food significantly changes prevalent expectations in the market; and entrepreneurs could benefit from addressing those demands. Similarly, the continuous development of many less-developed countries toward efficiency- or innovation-driven economies goes along with a rising middle class (for instance, in China), which dramatically changes demand in the food products sector.

**Table 1:** Classic economic sources of entrepreneurial opportunities

Source of entrepreneurial opportunity	Definition
Change in demand	Any change in customers’ demand provides entrepreneurial opportunity if entrepreneurs are able to cater to this demand
Change in supply	Changes in supply provide entrepreneurial opportunity by enabling entrepreneurs to rearrange the value chain
Information asymmetries	Reduction and/or creation of information asymmetries between supply and demand provide entrepreneurial opportunity
Exogenous shocks	Exogenous shocks such as new regulations or new technological solutions provide entrepreneurial opportunity by altering the mechanisms and/or frameworks of existing markets

Second, changes in supply, such as newly developed enzymes or flavorants, offer food entrepreneurs opportunities to remodel their value chain. Third, other potential drivers of entrepreneurial opportunities include information asymmetries that expose entrepreneurial opportunities for those entrepreneurs able to address them. Platforms educating consumers about food risk^12^ or matching consumers with local organic farmers offer examples of benefits accruing from an existing information asymmetry. Finally, exogenous shocks to the market are likely to present the most interesting entrepreneurial opportunities. Regulatory changes such as the European Union’s initiative to ban single-use plastics^13^ are an example of such shocks that dramatically alter existing markets. The EU initiative prompted a number of start-ups seeking products to substitute for single-use plastics (e.g., cutlery, plates, straws, stirrers, food containers, or cups for beverages) with edible alternatives.

## Entrepreneurial opportunities in the food value chain

The perception of entrepreneurial opportunities is highly subjective^14^ and hence we proxy for entrepreneurial opportunities in the food value chain by illustrating where exactly start-up investors allocate their investments. A high number of deals in a particular step of the food value chain would indicate investors perceive promising opportunities. We rely on the Dow Jones VentureSource database, which is one of the most comprehensive databases tracking the behavior of start-up investors, especially in Europe and the United States.^15^

For the years 2013 to 2017, Dow Jones VentureSource covers investments in food start-ups, particularly in its categories of *agriculture and forestry* and *food and beverages*. The database records 942 cases of investment for Europe and 1821 cases for the United States. Given that those two categories alone are not sufficiently informative, we reclassified those investments into a five-step-conceptualization of the food value chain inspired by Cugana and Goldsmith^16^ consisting of the steps: *agriculture*, *transforming*, *converting & packaging*, *shipping & selling* and, *consuming* (Table 2). Two researchers worked independently to assign all individual investment cases to a step in the food value chain. The coding illustrated substantial agreement among the raters.^17^ Contradictory codings were resolved with the help of a third rater.

**Table 2:** Five steps of the food value chain

Step	Definition
1. Agriculture	All activities and inputs required to cultivate crops and livestock
2. Transforming	Processing crops and livestock into food ingredients
3. Converting & Packaging	Composition of food products out of different ingredients and the transportation-ready packaging of the same
4. Shipping & Selling	Transportation, stocking and promotion of food to make it available for purchase
5. Consuming	Preparation of meals and provision of the same, e.g., in a restaurant or at home

Table 3 summarizes the results and compares the investment behavior of US and European investors in the food value chain. Overall, investment activity in the food value chain has been rising over the period of observation from 443 deals in 2013 to 747 in 2017, indicating an intensified interest in food start-ups. Most deals (62.69%) targeted the third step of the food value chain, namely *converting & packaging*, where products are composed from ingredients and packaged ready for transportation. The other four steps of the food value chain attract considerably less investment, with *shipping & selling* (transportation, stocking and promoting to make food available for purchase) accounting for 13.97% of the deals and *agriculture* (all activities required to cultivate crops and livestock) attracting 13.46% of the deals. *Transforming* (turning crops and livestock into food ingredients) and *consuming* appear to offer less interesting entrepreneurial opportunities, attracting only 5.68% and 4.20% respectively of investments in food start-ups. Given the typical investment time horizon of professional startup investors, which may approach up to 10 years,^18^ the data does not allow to provide answers whether this investment behavior is rationale and profitable. It is, however, striking to see that most deals take place in the middle of the food value chain, which is generally assumed^16^ to be its most profitable step.

**Table 3:** Investments in start-ups active in the food value chain (2013–2017)

Year	1. Agriculture			2. Transforming			3. Converting & Packaging			4. Shipping & Selling			5. Consuming			
	USA	Europe	Total	USA	Europe	Total	USA	Europe	Total	USA	Europe	Total	USA	Europe	Total	
	Deals (%)	Deals (%)	Deals (%)	Deals (%)	Deals (%)	Deals (%)	Deals (%)	Deals (%)	Deals (%)	Deals (%)	Deals (%)	Deals (%)	Deals (%)	Deals (%)	Deals (%)	Total year
2017	47 (8.92)	60 (27.27)	107 (14.32)	42 (7.97)	1 (0.45)	43 (5.76)	367 (69.64)	106 (48.18)	473 (63.32)	60 (11.39)	46 (20.91)	106 (14.19)	11 (2.09)	7 (3.18)	18 (2.41)	747
2016	17 (4.76)	48 (27.75)	65 (12.26)	25 (7.00)	9 (5.20)	34 (6.42)	256 (71.71)	76 (43.93)	332 (62.64)	34 (9.52)	36 (20.81)	70 (13.21)	25 (7.00)	4 (2.31)	29 (5.47)	530
2015	27 (8.26)	43 (22.28)	70 (13.46)	19 (5.81)	10 (5.18)	29 (5.58)	230 (70.34)	87 (45.08)	317 (60.96)	36 (11.01)	48 (24.87)	84 (16.15)	15 (4.59)	5 (2.59)	20 (3.85)	520
2014	31 (8.93)	26 (14.77)	57 (19.90)	10 (2.88)	21 (11.93)	31 (5.93)	253 (72.91)	92 (52.27)	345 (65.97)	35 (10.09)	26 (14.77)	61 (11.66)	18 (5.19)	11 (6.25)	29 (5.54)	523
2013	34 (12.93	39 (21.67)	73 (16.48)	15 (5.70)	5 (2.78)	20 (4.51)	165 (62.74)	100 (55.56)	265 (59.82)	38 (14.45)	27 (15.00)	65 (14.67)	11 (4.18)	9 (5.00)	20 (4.51)	443
Total	156 (8.57)	216 (22.93)	372 (13.46)	111 (6.10)	46 (4.88)	157 (5.68)	1271 (69.80)	461 (48.94)	1732 (62.69)	203 (11.15)	183 (19.43)	386 (13.97)	80 (4.39)	36 (3.82)	116 (4.20)	2763

*n* = 2.763 investments in the food value chain.

Comparing European and US investors reveals marked differences. US investors appear to focus on the middle of the food value chain by allocating the majority of their investments (69.80%) to the step of *converting & packaging*. Although European investors also allocate the bulk of their investments to this step (48.94%), they do so less intensely than their US counterparts and focus more on *agriculture* (22.93% European deals vs. 8.57% US deals) and *shipping & selling* (19.43% European deals vs. 11.15% US deals). Consequently, European investors, although less active overall, exhibit a more balanced approach to investing in entrepreneurial opportunities in the food value chain.

## Mapping the entrepreneurial opportunity space in the food value chain

**Fig. 1:**
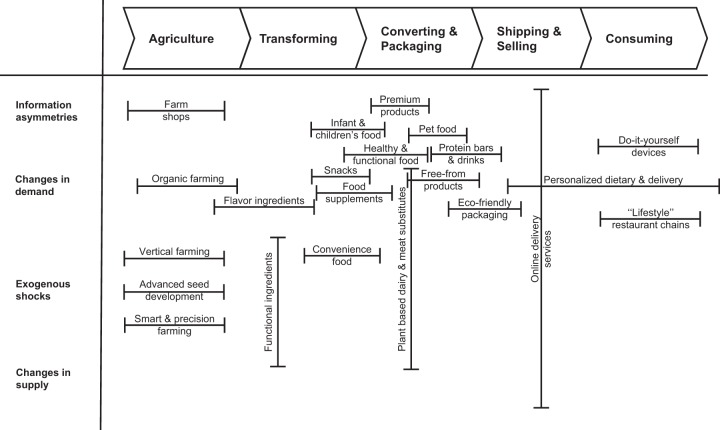
The entrepreneurial opportunity space in the food industry

Rigorously coding the start-ups in the sample to assign them to one of the nominated steps of the value chain created a profound knowledge of food industry firms’ value creation, which made it possible to reconstruct the entrepreneurial opportunity space in the food sector (Fig. 1) by mapping the four sources of entrepreneurial opportunities (i.e., *change in demand*, *change in supply*, *information asymmetries*, and *exogenous shocks*) against the five-step conceptualization of the food value chain ranging from *agriculture* to *consumption*. The resulting matrix of the opportunity space displays entrepreneurial opportunities within the food sector categorized by the conceptualized value chain of the food industry on the horizontal axis and by the four drivers of opportunities on the vertical axis. Each bar within the matrix represents an entrepreneurial opportunity within the food sector.

Three steps along the food value chain display interesting patterns. First, the two steps with the highest density of entrepreneurial opportunities are *converting & packaging* and *agriculture*. Second, *shipping & selling* reveals an opportunity facilitated by all four sources of opportunity simultaneously. Given that these steps of the food value chain were already identified as the steps attracting the most investments, it is not surprising to see that they display the largest variety of opportunities. However, there is value in scrutinizing the combination of the food value chain steps and the respective sources of entrepreneurial opportunity.

As the entrepreneurial opportunity space illustrates, opportunities within *converting & packaging* are mainly driven by *change in demand*, whereas the main sources of opportunities within *agriculture* seem to be related to *exogenous shocks* to the market such as global population growth or climate change that call for more efficiency in agricultural production. Opportunities emerging from such shocks include smart farming, which would encompass initiatives such as using sensors and the analysis of big data to deliver the precise application of fertilizers and herbicides, and vertical farming, which targets overcoming the spatial constraints of conventional farming. Moreover, development of pathogen-free and robust seed using modern genome editing techniques paves the way for disease- or drought-resistant plant varieties.

The *converting & packaging* step offers a plethora of opportunities owing to a *change in demand*. This step of the food value chain holds potential for entrepreneurs seeking to differentiate themselves from their competitors. The rising awareness of health issues when combined with the growing proportion of the population reporting food allergies facilitates a complete sector branch dedicated to so-called *free from* products, but also for food supplements, fitness protein drinks, or power bars. Alongside increasing wealth among consumers, opportunities arise in niche markets such as that for pet food as well.

But as the example of plant-based meat and dairy substitutes shows, opportunities are not always facilitated by only one source. Currently, there is widespread recognition of the need for input reduced production to reflect the issues of climate change and population growth (*exogenous shocks*), an increasing awareness of environmental pollution and animal welfare (*change in demand*), and of new technological abilities in biotechnology and process engineering (*change in supply*), the combination of which fosters opportunities in this emerging sector branch.

An even more extreme constellation covering all four drivers is observable for online delivery services within the step of *shipping & selling*. Digitization, which could be interpreted as an *exogenous shock* in many markets, makes it possible for entrepreneurs to offer customized and individually delivered products (*change in supply*), thereby meeting consumer demands for more flexibility (*change in demand*) and to combine supply and demand via online platforms in an as yet unseen manner in order to mitigate *information asymmetries*. This diverse set of sources of opportunity enables entrepreneurs to devise many different solutions and to shape their business models by adjusting the emphasis on value creation to target many different customer segments.

## Conclusions and recommendations

Any situation “in which new goods, services, raw materials, and organizing methods can be introduced and sold at greater than their cost of production”^8^ constitutes an entrepreneurial opportunity and the current analysis has uncovered a plethora of such situations, although the suggested categorization might not be completely disjunct. Still, the food industry provides various entrepreneurial opportunities along the food value chain as the suggested map of the opportunity space illustrates. Following the investor perspective, *converting & packaging*, *agriculture*, and *shipping & selling* are the steps of the food value chain attracting the most funding. This is an indication that these are generally favorable areas for entrepreneurial activity in the food value chain.

Moreover, given that investors tend to specialize, and that it will therefore be challenging for an aspiring food entrepreneur to convince an investor focusing on one step of the food value chain to invest in an alternative step, funding a food start-up targeting the most prominent steps of the food value chain promises to be less challenging for entrepreneurs. However, there are several areas within the opportunity space that have yielded only a small number of opportunities or that appear to yield none at all. Given that the most interesting start-ups of the past in any industry were highly innovative and differentiated themselves almost completely from the existing players in the market, these blank spaces might in fact constitute the most interesting areas for aspiring food entrepreneurs, supposing that they perceive a truly innovative solution to a pressing customer problem in these areas and that their solution can create real value.

### Reporting Summary

Further information on research design is available in the Nature Research Reporting Summary linked to this article.

## Supplementary information

Reporting summary

## Data Availability

Dow Jones Venture Source is a proprietary global database on venture capital and private equity backed companies available at https://www.dowjones.com/products/pevc/.

## Supplementary information

**Supplementary information** accompanies the paper on the *npj Science of Food* website (10.1038/s41538-019-0039-7).
